# Quantifying and Predicting the Effect of Exogenous Interleukin-7 on CD4^+^T Cells in HIV-1 Infection

**DOI:** 10.1371/journal.pcbi.1003630

**Published:** 2014-05-22

**Authors:** Rodolphe Thiébaut, Julia Drylewicz, Mélanie Prague, Christine Lacabaratz, Stéphanie Beq, Ana Jarne, Thérèse Croughs, Rafick-Pierre Sekaly, Michael M. Lederman, Irini Sereti, Daniel Commenges, Yves Lévy

**Affiliations:** 1 INSERM, ISPED, Centre INSERM U897-Epidemiologie-Biostatistique, Bordeaux, France; 2 Univ. Bordeaux, ISPED, Centre INSERM U897-Epidemiologie-Biostatistique, Bordeaux, France; 3 INRIA, SISTM team, Bordeaux, France; 4 Laboratory for Translational Immunology, University Medical Center Utrecht, Utrecht, The Netherlands; 5 Theoretical Biology and Bioinformatics, Department of Biology, Utrecht University, Utrecht, The Netherlands; 6 INSERM, Unité U955, Créteil, France; 7 Université Paris-Est, Faculté de Médecine, UMR-S955 Creteil, France; 8 CYTHERIS, Issy Les Moulineaux, France; 9 Vaccine and Gene Therapy Institute-Florida, Port St. Lucie, Florida, United States of America; 10 Case Western Reserve University/University Hospitals/Case Medical Center, Cleveland, Ohio, United States of America; 11 National Institute of Allergy and Infectious Diseases, National Institutes of Health, Bethesda, Maryland, United States of America; 12 AP-HP, Groupe Henri-Mondor Albert-Chenevier, Immunologie Clinique, Creteil, France; Imperial College London, United Kingdom

## Abstract

Exogenous Interleukin-7 (IL-7), in supplement to antiretroviral therapy, leads to a substantial increase of all CD4^+^ T cell subsets in HIV-1 infected patients. However, the quantitative contribution of the several potential mechanisms of action of IL-7 is unknown. We have performed a mathematical analysis of repeated measurements of total and naive CD4^+^ T cells and their Ki67 expression from HIV-1 infected patients involved in three phase I/II studies (N = 53 patients). We show that, besides a transient increase of peripheral proliferation, IL-7 exerts additional effects that play a significant role in CD4^+^ T cell dynamics up to 52 weeks. A decrease of the loss rate of the total CD4^+^ T cell is the most probable explanation. If this effect could be maintained during repeated administration of IL-7, our simulation study shows that such a strategy may allow maintaining CD4^+^ T cell counts above 500 cells/µL with 4 cycles or fewer over a period of two years. This in-depth analysis of clinical data revealed the potential for IL-7 to achieve sustained CD4^+^ T cell restoration with limited IL-7 exposure in HIV-1 infected patients with immune failure despite antiretroviral therapy.

## Author Summary

HIV infection is characterized by a decrease of CD4^+^ T-lymphocytes in the blood. Whereas antiretroviral treatment succeeds to control viral replication, some patients fail to reconstitute their CD4^+^ T cell count to normal value. IL-7 is a promising cytokine under evaluation for its use in HIV infection, in supplement to antiretroviral therapy, as it increases cell proliferation and survival. Here, we use data from three clinical trials testing the effect of IL-7 on CD4^+^ T-cell recovery in treated HIV-infected individuals and use a simple mathematical model to quantify IL-7 effects by estimating the biological parameters of the model. We show that the increase of peripheral proliferation could not explain alone the long-term dynamics of T cells after IL-7 injections underlining other important effects such as the improvement of cell survival. We also investigate the feasibility and the efficiency of repetitions of IL-7 cycles and argue for further evaluation through clinical trials.

## Introduction

Human Immunodeficiency virus (HIV) infection is characterized by a profound depletion of CD4^+^ T cell numbers and function. Immune restoration with combination antiretroviral therapies (cART) has substantially improved patients' outcomes. Unfortunately, this restoration may be delayed, notably in patients starting treatment late, and/or incomplete, despite control of the viral replication [1]. Hence, immune therapy may be a complementary intervention to accelerate or improve immune restoration. Interleukin-7 (IL-7) is a cytokine produced by non–marrow-derived stromal and epithelial cells and is required for the development and persistence of T cells in the periphery [2], [3]. IL-7 may enhance thymopoiesis [4]–[6], as well as thymic-independent peripheral proliferation of recent thymic emigrants [7]–[9] and of more mature T cells [7], [9] even in the absence of cognate antigen [9]–[11]. Improved cell survival has also been shown *in vivo* [11]–[15]. In HIV-infected patients, a strong inverse correlation has been observed between plasma IL-7 levels and CD4^+^ T cell numbers as well as with CD4^+^ T cell reconstitution after initiation of antiretroviral therapy [16], [17]–[19]. Increased levels of IL-7 during lymphopenia are thought to be mainly the consequence of a decreased receptor-mediated clearance of IL-7 as the availability of receptors diminishes [20]. In addition, the IL-7 signaling on IL-7 receptor-a-positive (IL-7Ra+) dendritic cells in lymphopenic settings may diminish the homeostatic proliferation of CD4+ T cells [21]. A recent study has suggested that the remaining chronic inflammation in treated HIV-infected patients due to exposure to IL-1β and IL-6 may decrease T-cell sensitivity to IL-7 and therefore a reduced CD4+ T cell reconstitution [22]. Recent analyses of lymph node tissues have shown that collagen deposition may restrict T-cell access to IL-7, resulting in apoptosis and depletion of T cells [23]. This in turn leads to decreased production of lymphotoxin B, a trophic factor for reticuloendothelial cells, leading to their demise and loss of IL-7 producing cells. In summary, the IL-7 effect on CD4+ T-cell homeostasis is highly compromised in HIV infection [24].

The beneficial effect of administration of IL-7 on T cell homeostasis in patients with refractory cancer [9], in SIV infected macaques [15], [25], [26] and HIV infected individuals has been shown through several early trials [27], [28] and observational studies [29]. However, before proceeding with phase II and III trials, several questions remain. From a mechanistic standpoint, the respective contributions of thymic production [9], peripheral proliferation and survival [11] in the observed increase of CD4^+^ T cell count in HIV-infected patients are unclear. Also, the schedule of IL-7 administration, notably the frequency of cycling needed to reach optimal and durable CD4^+^ T cell restoration is not defined. Finally, the long-term effects of IL-7 therapy and repeated IL-7 cycles on T cell homeostasis in HIV-infected patients are unknown. To address these questions, we have developed a mathematical model to approximate the effect of IL-7 on CD4^+^ T cell homeostasis to fit the data from two phase I trials of IL-7 intervention in HIV-infected patients. This analysis is most consistent with a significant additional biological effect (on cell survival and/or thymic production) to the observed transient increase in peripheral cell proliferation. The predictions from the model have been used to explore the feasibility of repeated “maintenance” cycles of IL-7 administration with the aim of maintaining a given level of circulating CD4^+^ T cells.

## Results

### IL-7 induced a sustained increase of all CD4^+^ T cell subsets

**Figure 1:**
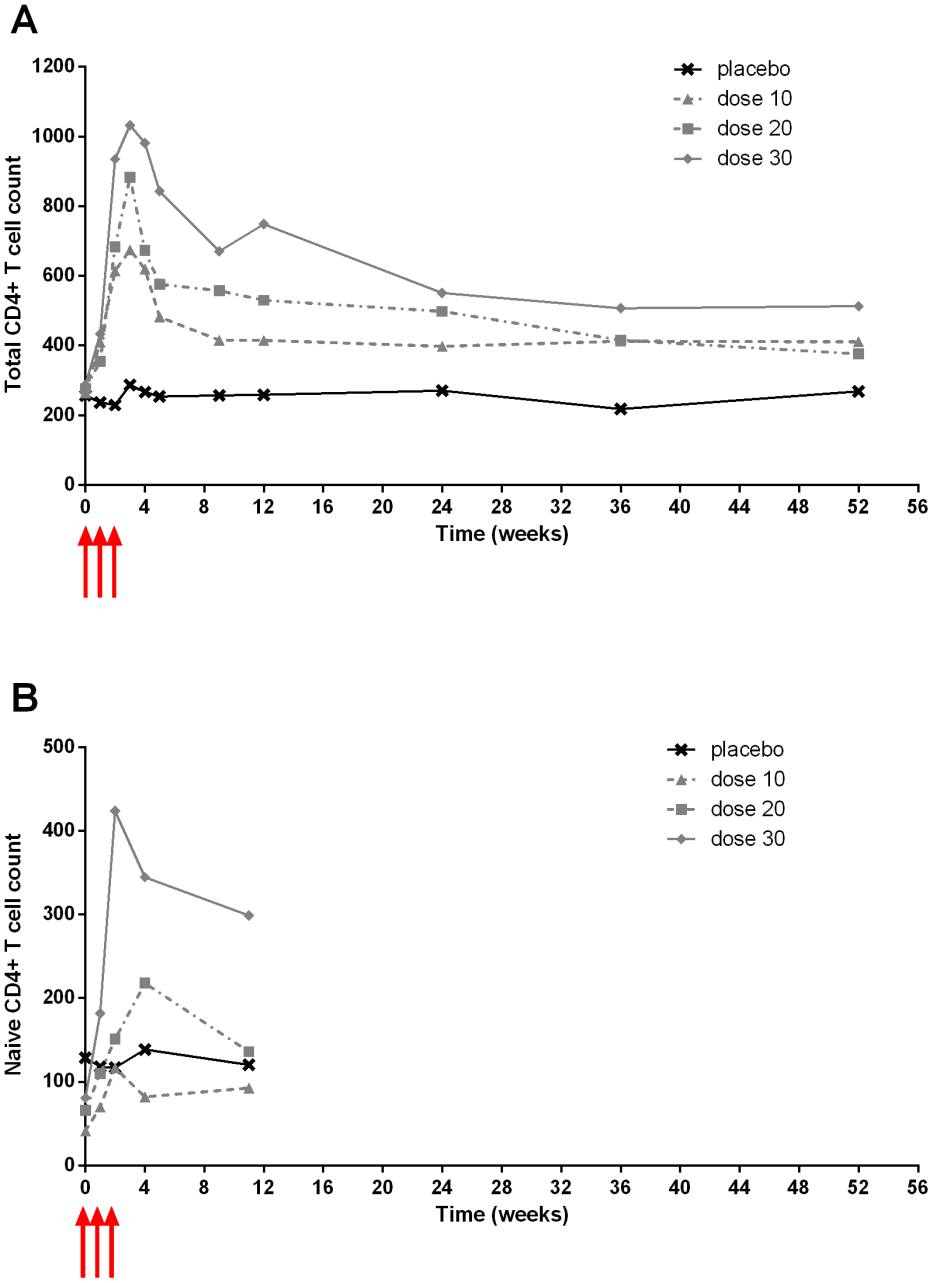
Dose-dependent increase of total CD4^+^ T cell count (A) and naive (CD45RA^+^CD27^+^CCR7^+^) CD4^+^ T cell count (B) for INSPIRE Study (Study II). Observed median count in cells/µL by group: placebo (crosses), 10 µg/kg (triangles), 20 µg/kg (squares) and 30 µg/kg (diamonds). Red arrows indicate IL-7 administration. Error bars and other statistical analyses are provided in Levy et al. [30].

**Figure 2:**
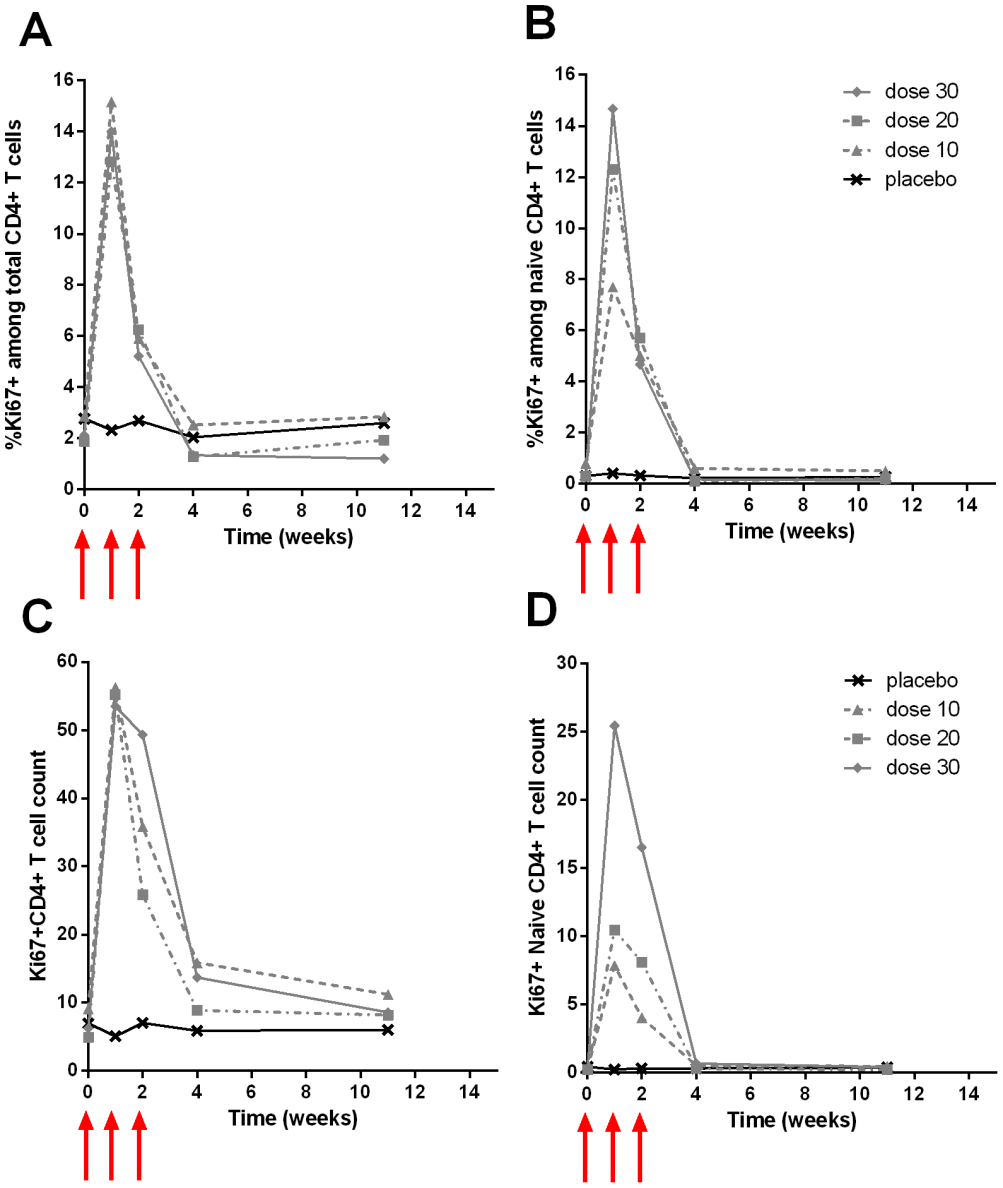
Increase of percentage of Ki67^+^ among CD4^+^ T cells (A), naive (CD45RA^+^CD27^+^CCR7^+^) CD4^+^ T cells (B); total CD4^+^Ki67^+^ cell counts (C) and naive CD4^+^Ki67^+^ cell counts (D) for INSPIRE Study (Study II) depending of the dose. Observed median percentage of Ki67^+^ among CD4^+^ T cells by group: placebo (crosses), 10 µg/kg (triangles), 20 µg/kg (squares) and 30 µg/kg (diamonds). Red arrows indicate IL-7 administration. Error bars and other statistical analyses are provided in Levy et al. [30].

Chronically HIV-1 infected patients with CD4^+^ T cell counts between 100 and 400 cells/µL and plasma HIV RNA<50 copies (c)/mL while on antiretroviral therapy were studied in three phase I/II trials (see Methods and Table 1 for characteristics). In Study I and II, there was a dose-dependent increase of CD4^+^ T cell count peaking between 14 and 21 days after the initial injection and followed by a steady decline. The peak increase ranged between 152 and 1202 CD4^+^ T cells/µL in the two studies [28], [30]. A significant increase compared to baseline (and placebo group in Study II) persisted until 12 weeks in the first study (Figure S1) and 52 weeks in the second (Figure 1). The main contributors to CD4^+^ T cell increase were naive and central memory cells [28], [30]. There was a transient increase of Ki67 expression (a marker of proliferation; see Methods) in all CD4^+^ T cell subsets during IL-7 administration (Figure 2). The peak of Ki67 expression was observed at the first available measurement after the initiation of IL-7 therapy, which is 14 days in study I and 7 days in study II. At 28 days, Ki67 expression returned to baseline in both studies.

**Table 1:** Characteristics of phase I/II trials and patients included from each study.

Characteristics	rh-IL-7 study [28]	INSPIRE [30]	INSPIRE 2
**Trial number**	2004-003772-11A	NCT0047732	NCT01190111
**Product**	rh-IL-7 CYT 99 007	Glyco r-hIL-7 CYT107	Glyco r-hIL-7 CYT107
**Doses**	3, 10 µg/kg	10, 20, 30 µg/kg	20 µg/kg
**N patients enrolled**	14	26 (+6 placebo)	23
**N patients analyzed** *	14	21 (+6 placebo)	12
**Number of measurements of CD4 T cells per patient** **	11	11	5
**Number of measurements of Ki67+ CD4 T cells per patient** **	4	5	5
**Number of measurements of naive CD4 T cells per patient** **	Na	5	Na
**Number of measurements of Ki67+ naive CD4 T cells per patient** **	Na	5	Na
**Baseline CD4 T cells/µL** **	230 (107; 312)	276 (231; 320)	246 (200; 318)

* Patients with complete cycles used for the model.

** Median (Interquartile range).

Na: Not applicable.

This increase in cell proliferation, observed in parallel with the increase in CD4^+^ T cells, might be the only significant effect *in vivo* of the injection of exogenous IL-7. Indeed, IL-7 induces an acute cellular proliferation during a short time period leading to a rapid CD4+ T cell increase, followed by a slow return to baseline levels as CD4^+^ T cells die, explaining the observed dynamics. However, additional effects, especially on thymic output or cell survival, might exist and slow down the decline of CD4^+^ T cells. Recent thymic emigrants (defined as CD45RA^+^CD31^high^) and the sj/β T cell receptor excision circles (TREC) ratio (in Study II), which are both an indirect measure of thymic output [31], are significantly increased after IL-7 injections [28], [30]. In Study II, we also observed a decrease in PD-1 expression (a marker of cell exhaustion) by CD4^+^ T cells [30] suggesting an increased cell survival. Although these observations gave some insight in potential effects of IL-7 on T cell homeostasis, they do not quantify the respective contribution of these mechanisms to the observed CD4^+^ T cell dynamics in blood in terms of input and output of cells. This is why we embarked on a mathematical analysis to test whether the observed peripheral proliferation could explain the CD4^+^ T cell dynamics after IL-7 injections or if other additional biological mechanism played a significant role.

### Mathematical modeling revealed that total CD4^+^ T cell dynamics are a consequence of more than a transient increase of peripheral cell proliferation

**Figure 3:**
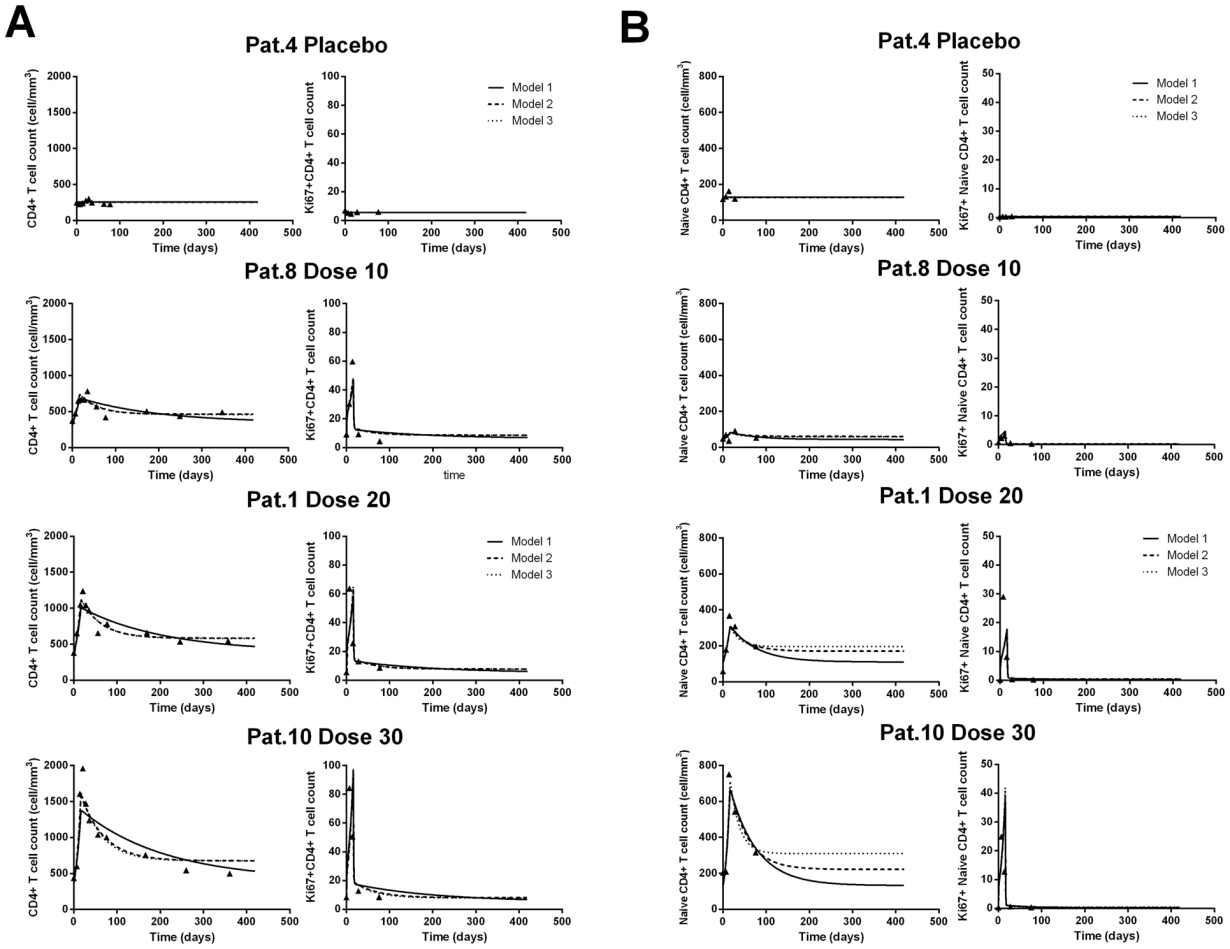
Goodness of fit of total (A) and naive (B) CD4^+^ T cell count for a random patient in each dose group from INSPIRE Study (Study II) for the three different models. The prediction from model 1 assuming only an effect of IL-7 on the proliferation rates is in solid line, from Model 2 assuming an effect on proliferation rate and on the loss rate of resting cells in dashed line and from Model 3 assuming an effect on proliferation rate and on the thymic production in dotted lines. Note that the estimated trajectories from Model 2 and 3 almost overlap.

**Figure 4:**
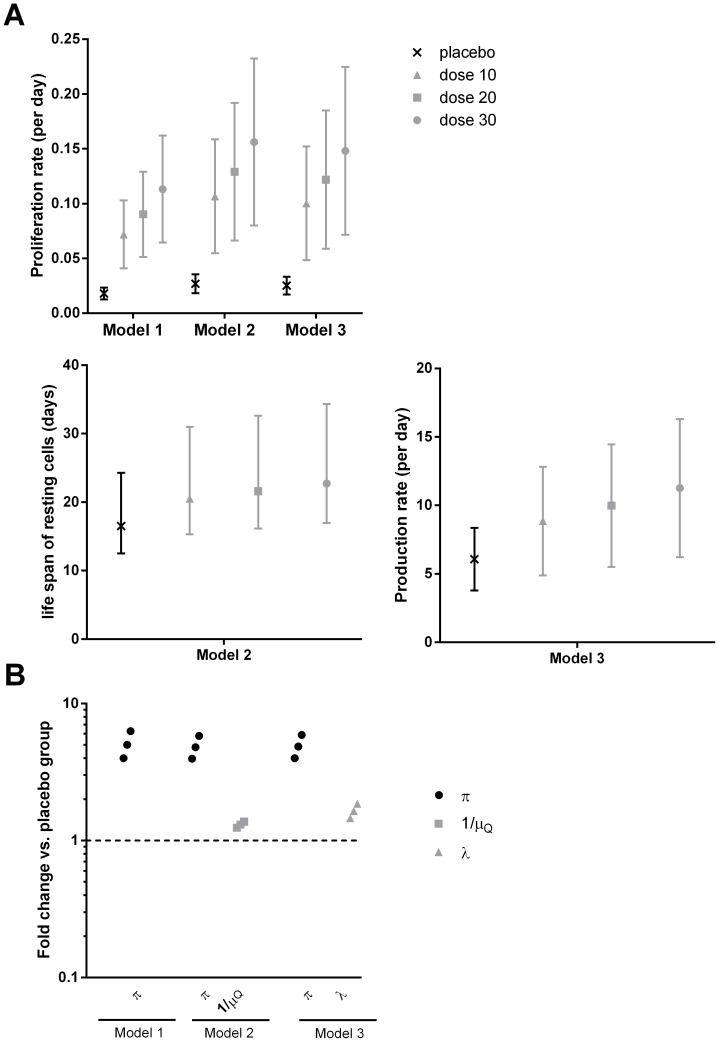
Estimated effects of IL-7 injection on the biological parameters for total CD4^+^ T-cells in Study II. Parameter estimates of Model 1 (effect on proliferation rate only), Model 2 (effect on proliferation and loss rate of quiescent cells) and Model 3 (effect on proliferation and production) with 95% Confidence Intervals for total CD4+ T-cells in INSPIRE Study (A). Fold change in proliferation rate (π) during IL-7 administration, life-span (1/μ_q_) and production rate (λ) after IL-7 administration in each dose group compared to the placebo group in INSPIRE Study (Study II) for total CD4^+^ T-cells (B).

We used a simple mathematical model to investigate mechanistically the effect of IL-7 on total CD4^+^ and naive CD4^+^ T-cell dynamics (see Methods and Figure S2). Modeling CD4^+^ dynamics and Ki67^+^ expression by changing the proliferation rate during IL-7 administration provided a fair fit of the data of study II (Figure 3A, plain lines). Interestingly, there was a significant linear increase of estimated proliferation rates according to the dose group (p<0.0001; Figure 4A and 4B). However, we found a better fit of CD4^+^ dynamics with Model 2 (LCVa −0.173 vs. 0.937; Figure 3A, dashed lines) that includes an effect of IL-7 on proliferation rate during IL-7 administration and on loss rate after IL-7 administration. In addition to the significant dose-dependent increase of proliferation during IL-7, we estimated a decrease of the loss rate of quiescent cells from 0.061 to 0.044–0.049 per day corresponding to an improvement of the life span of about 25% from 16.4 days to 20.4–22.7 days (likelihood ratio test p-value<0.001; Figure 4, Table 2 and Table S1). This result was found with both formulation of IL-7 (with either rh-IL-7 or glycosylated rh-IL7; Table S2). Adding a modification of the constant production of CD4^+^ (Model 3) rather than a modification of loss rate (Model 2) did not substantially improve the fit to total CD4+ T cell dynamics as shown in Figure 3A where the fits from the two models overlap (see also Figure S3 and S4). In other words, although the Model 2 that includes a modification of quiescent cell loss rate was better from a statistical point of view (LCVa = −0.131 vs. −0.173), it was difficult to distinguish the fits of the two models. Interestingly, all models described correctly the initial increase of CD4+ T cells and thereafter, Model 1 predicted a slower decline of CD4+ T cells than Model 2 and 3. This poorer long-term fit might be explained by a transient effect on the proliferation rate (until day 16) that altered only briefly the equilibrium while the lingering effect on the production or loss rate changed it in the long-term.

**Table 2:** Estimates of the “best” model for total CD4^+^ and naive CD4^+^ T-cell dynamics in Study II (INSPIRE Study).

			Total CD4	Naive CD4
**Production Rate**	before & during IL7		9.849 (2.126)	***3.600 (1.518)***
**(λ, cells/day)**	after IL7	10 µg/kg	9.849	***4.904 (2.883)***
		20 µg/kg	9.849	***6.619 (3.347)***
		30 µg/kg	9.849	***8.935 (3.885)***
**Proliferation Rate**	before & after IL7		***0.027 (0.004)***	***0.004 (0.001)***
**(π, /day)**	during IL7	10 µg/kg	***0.107 (0.026)***	***0.068 (0.003)***
		20 µg/kg	***0.129 (0.032)***	***0.105 (0.003)***
		30 µg/kg	***0.156 (0.039)***	***0.161 (0.004)***
**Loss rate of non- proliferating cells**	before & during IL7		***0.061 (0.010)***	0.050 (0.019)
**(μ_Q_, /day)**	after IL7	10 µg/kg	***0.049 (0.008)***	0.050
		20 µg/kg	***0.046 (0.008)***	0.050
		30 µg/kg	***0.044 (0.007)***	0.050
**Loss rate of proliferating cells (μ_P_, /day)**			0.070 (0.031)	0.078 (0.039)
**Reversion rate to quiescent state (ρ, /day)**			1.213 (0.173)	1.199 (0.255)
**σ_λ_** *			−0.205 (0.066)	0.523 (0.132)
**σ_ρ_** *			0.396 (0.165)	0.458 (0.308)

Bold-italic numbers represent significant IL-7 effects. Standard-errors are given between brackets. Parameters for the other models and other studies are presented in Supplemental Materials.

* Standard-deviation of random effect.

Note that the random effects were on the log-transformed parameter and not on the natural scale.

To further analyze the potential effect of IL-7 on thymic output, we explored the effect of IL-7 on the naive (CD45RA^+^CD27^+^) CD4^+^ cells (either Ki67^+^ or Ki67^−^) using the available data for this subset (until 12 weeks). Here again, we found that an additional effect of IL-7 after its administration either on the thymic production or the loss rate significantly improves the fits compared to a model including only an effect on the proliferation rate (Table S3). Interestingly, we found that the best model was the one including an effect on the thymic production rate of naive cells after IL-7 administration in addition to the proliferation rate (LCVa = 1.705 vs. 1.760 for the model including an effect on the loss rate after IL-7 administration; Table 2 and Table S3). Both models including an additional effect of IL-7 were better than the model with an effect on proliferation only (LCVa = 1.832). However, a change in loss rate of quiescent cells led to a good fit as well and individual fits from Model 2 and 3 were very close as shown in Figure 3B and S5.

Finally, we were interested in the ability of Model 2 (with the effect of IL-7 on proliferation and loss rates) to predict individual responses to IL-7. We made use of data from 12 additional patients (from INSPIRE 2, Table 1) treated with a 20 µg/kg dose as per the INSPIRE study. We used only the first two measurements of total CD4^+^ T cells and Ki67^+^ cells to compute the Empirical Bayes estimates for each parameter that could vary between patients. The other population parameters were fixed according to the previous estimations (Table S1). We then predicted the individual CD4^+^ T cell dynamics until week 12. Most of the observed total CD4^+^ T cell counts were in the prediction interval (Figure S6). Therefore, the model that includes an effect of IL-7 on proliferation and loss rates led to a good description of the total CD4^+^ T cell dynamics and a fair predictive ability at the individual level.

### Potential for IL-7 to achieve sustained CD4 restoration with repeated administration

To our knowledge, no data exists yet on the effect of repeated cycles of IL-7 *in vivo*. Therefore, to investigate to what extent IL-7 administration might sustain CD4+ T-cell restoration, we artificially created data and compared different scenarios allowing the IL-7 effect to wane after subsequent injections compared to the initial one (see Methods). In this part, we considered an extended version of the mathematical model that incorporates a homeostatic proliferation (see Methods). This model gave similar results as presented in the previous section (not shown) but more realistic long-term dynamics for repeated IL-7 administrations (total CD4^+^ T cell counts staying below 1500 cells/µL).

As the dose 20 µg/Kg was the one recommended for further phase II/III studies [30], we simulated CD4+ T cell dynamics with hypothetical repeated cycles of IL-7 administration for each patient who received this dose in the Study I (INSPIRE): namely 14 patients. The parameter *κ* controlling the proliferation rate was fixed to the same value for each patient (see Methods). The initial CD4+ dynamics was the one predicted by the Model 2 as presented in the previous section. CD4^+^ T cell count were assumed to be measured every three months and when it dropped below 500 cells/µL a cycle of IL-7 administration was simulated (one injection per week for 3 weeks). The dynamics were therefore based in part on fit to observed data and in part on a predicted response to repeated therapy. We analyzed two primary outcomes: the time spent above 500 cells/µL and the number of cycles (including the first cycle) needed to maintain CD4^+^ T cell counts above 500 cells/µL over 2 years. A secondary outcome was the median time between two successive cycles.

**Figure 5:**
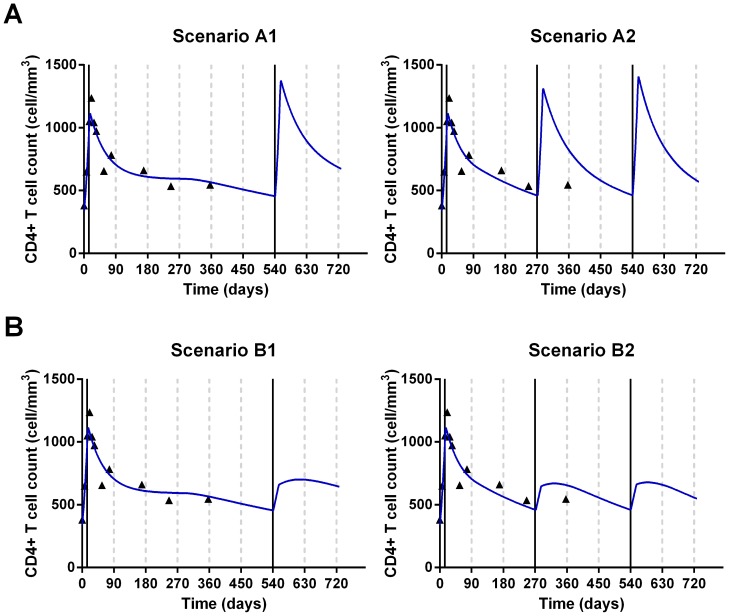
Simulated trajectories of CD4^+^ T cell dynamics over 2 years for patient #1 with repeated IL-7 injections. Simulated treatment is based on CD4+ T cell count measurements (Triangles are the observed values) every three months (grey dashed vertical lines). If the count drops below 500 cells/uL, a new cycle of three IL-7 injections is simulated (single black vertical lines). A. Effect on both proliferation and loss rate of non-proliferating cells was full and constant during subsequent IL-7 cycles (scenario A-1 and A-2, see Table S4). B. The effect on proliferation during repeated cycles was assumed to be 50% of the one of the first cycle while the effect on loss rate was unchanged (scenario B-1 and B-2, see Table S4). Other parameters are presented in Table S1 (Model 2, dose 20), *κ* = 0.176.

Simulations were performed according to several scenarios varying from a constant effect after each cycle and decreasing effects on loss and proliferation rates. At each cycle, we assumed that the effect of IL-7 on the loss rate of CD4^+^ T cells started to decrease 3 (or 9) months after the last injection and disappeared after 1 (or 2) year. Table S3 shows some scenarios ordered from the best to the worst according to the primary outcomes. Where all the IL-7 cycles were assumed to keep 100% of their effects on CD4^+^ T cell counts (Scenario A-1 and A-2; Figure 5A and Table S4), the intervention was highly effective. CD4^+^ T cell counts were maintained above 500 cells/µL between 84% and 91% of the time compared to 11.5% when no new IL-7 cycle was administrated during the 24 months of follow-up. Moreover, the time between two cycles of IL-7 was estimated to be greater than 6 months. We also investigated reduced effect of IL-7 on peripheral proliferation and/or loss rate of non-proliferating cells during subsequent cycles. The scenario assuming that during repeated cycles only 50% of the effect on proliferation was effective while the effect on the loss rate was conserved (scenario B-1 and B-2; Figure 5B and Table S4) gave similar results as the scenario assuming a full and constant effect of proliferation and loss rate (Scenario A-1 and A-2; Table S4). Moreover, we observed that the more the effect of IL-7 on the loss rate was reduced, the more often cycles have to be administrated and the shorter the time between two successive cycles (Figure S7). These results suggest that it is more important to keep a strong effect on the loss rate of non-proliferating cells than on proliferation. As shown in Figure S7, when the effect of IL-7 is only maintained on the proliferation rate (i.e. no more effect in the loss rate of non-proliferating cells: α_μQ_ = 0), we predicted the highest number of injections, whatever the size of the effect on the proliferation rate, to sustain CD4 count above 500 cells/µL. Overall, in comparison to no repeated cycle (Reference scenario in Table S4) whatever the assumed effect of IL-7 during successive cycles, the CD4^+^ T cell count may be durably increased with clinically realistic administration schedules. Therefore, IL-7 repeated cycles seems feasible and efficient.

## Discussion

We report here a mathematical analysis of total and naive CD4^+^ T cell dynamics in HIV-1 infected patients treated with antiretrovirals who experienced a significant increase of CD4^+^ T cell counts while receiving IL-7 therapy. We confirm, once again, that IL-7 induces a significantly increased peripheral proliferation of CD4^+^ T cells as measured by Ki67 expression. However, results presented here extend our knowledge on the *in vivo* effects of IL-7 by showing that this increased peripheral proliferation alone could not explain the long-term changes in CD4^+^ T cell number that were observed. An increase of the production rate of naive CD4^+^ T cells and a decrease of the loss rate for total CD4^+^ T cells might also contribute to T-cell homeostasis during IL-7 therapy. Importantly, baseline parameters such as naive T cell production (around 9×10^8^ naive cells/day) [32], loss rate of proliferating cells (0.08 day^−1^) [33] or reversion rate (accounting for duration of division and duration of Ki67^+^ expression) were in agreement with current knowledge. Furthermore, our mathematical model shows good performance for individual predictions and provides insights on the feasibility of repeated cycles of IL-7 (or long-lasting formulation) for maintaining CD4^+^ T cell counts in HIV-infected patients. Predictions from the mathematical model underline the importance of an additional effect of IL-7 beyond peripheral cell proliferation for long-term CD4^+^ T cell responses.

HIV infection leads to a profound disturbance of T cell homeostasis with an increased turnover of these cells [33]–[35]. The naive T cell pool is replenished mainly by post-thymic proliferation in adults [36], [37] but the observed proliferation of naive CD4^+^ T cells is not enough to prevent the slow decline of these cells in HIV infected patients. Augmenting immunity with exogeneous cytokines has been attempted in HIV infection; and although CD4 T cell numbers were increased with administration of IL-2, two large clinical trials failed to show any evidence of clinical benefit [38]. The failure of IL-2 therapy to confer clinical benefit despite CD4 T cell increases could be attributed at least in part to the regulatory phenotype of the expanded cells [39] and the possibility of enhanced inflammation and coagulation during administration [40]. The effects of IL-7 on the other hand are fundamentally different [13] and the defined role of IL-7 in maintaining T cell homeostasis in health provides rationale for testing its therapeutic administration in HIV infection complicated by immune failure [41].

Our data argue for an increase in cell survival after IL-7 administration. Our results show that CD4^+^ T cell dynamics are better explained by a decrease of cell loss in addition to the transient peripheral proliferation. This finding is consistent with previous findings that increasing cellular survival through up-regulation of bcl2 expression is a physiological function of IL-7 [13], [42]. Moreover, increases in T cell survival after IL-7 injection have been demonstrated in monkeys using BrdU labeling [15]. Our findings warrant further study to define the precise mechanisms of IL-7 induced cell expansion in HIV infection.

Improvement of thymopoiesis has been reported during exogenous IL-7 administration [5], [6]. However, there is some controversy on the importance of this effect *in vivo* [9], [43]. Sportes et al. showed a modest increase of absolute numbers of TRECs and a major dilution of TREC content due to peripheral cell proliferation leading to the conclusion that the increase of TCR repertoire diversity is mainly due to the proliferation of recent thymic emigrants. The effects of IL-7 on thymopoiesis may be dependent, on one hand, on the underlying disease (HIV-1 infection, cancer and chemotherapy) and, on the other hand, on the duration of cytokine therapy [9]. In our simulations of repeated IL-7 cycles, we did not consider any effect of IL-7 on thymopoiesis because we favoured the hypothesis of an effect on cell survival according to the rationale above and the slightly better fit. Therefore, if IL-7 administration improved thymopoiesis in addition to peripheral proliferation and enhanced cell survival, the CD4^+^ T cell response should have surpassed our predictions.

For long-term predictions using simulations, the initial model was extended by adding a homeostatic control of proliferation after IL-7 administration. This can be related to the modulation of IL7Ra expression that prevents uncontrolled proliferation [44], [45]. Furthermore, the effect on T cell loss was also modeled to wane over time. Strikingly, the estimation of the duration of the effect of IL-7 on cell survival was prolonged up to 2 years. Likely, this could not be explained by the pharmacokinetics of exogenous IL-7 that was administrated during two weeks only. However, exogenous IL-7 is known to bind to components of the extracellular matrix resulting in saturation of this tissue compartment followed by a slow release of IL-7, which may exert long-lasting effects [3]. Also, IL-7 could have persistent effects on cellular homeostasis by normalizing tissue architecture through decreasing fibrosis in the gut [46] and in lymph nodes [23], [47], [48], [49] and thus improving cellular access to survival signals. Other potential activities such as effects on cell trafficking [27], [50], IL-7 antibody formation, switch to memory phenotypes [14], [51] or impact on proviral HIV DNA content [52] have not been taken into account in this model. Redistribution of CD4^+^ T cells to tissues, leading to a transient decrease of CD4^+^ T cells levels in blood, is mainly observed in the first days after IL-7 administration and should not affect measurements made thereafter. Although neutralizing anti-IL-7 antibodies were not observed in patients following the first cycle of IL-7 [28], their induction after repeated administration could attenuate the effects that we modeled here. For these reasons, new clinical trials are needed to help distinguishing the persistent IL-7 effect(s) involved in CD4 recovery in HIV-infected patients and to propose personalized therapy in the future [53]. Furthermore, we assumed repetition of cycles (i.e. three injections over two weeks) but the repetition of single injections could lead to similar results shown in the simulations if the effect of one injection respect the assumptions made in some scenarios. For instance, the repetition of a single injection may have a reduced effect on proliferation and cell survival compared to a whole cycle but this would still lead to a good maintenance of CD4^+^ T cell counts.

There are limitations to this study that include the restricted number of harvest times and the lack of validated markers for cellular lifespan. One way to overcome these limitations and to help distinguishing between increased production and increased survival is to perform studies that include *in vivo* labeling with deuterium and TREC content measurements. Indeed, deuterium labeling is a recent and powerful tool to estimate cell turnover [54] and used in combination with TREC content allows estimation of thymic output [37], [55]. Also, it may have been relevant to distinguish the dynamics in lymph node tissue to better capture long-term effect of IL-7 although data on lymph node tissue would be difficult to obtain. Despite these caveats, the goodness of fit and the predictive capacity of the model provide important insights for further development of IL-7 treatment strategies. We have learned that IL-7 administration leads to a burst of peripheral proliferation that is likely associated with a lingering effect beyond the period of IL-7 administration. We surmise that a durable effect of IL-7 on T cell homeostasis could be achieved after repeated administration but safety and activity need to be confirmed [2], [3].

## Materials and Methods

### Subject population and trial design

Data were generated in three phase I/II studies (Table 1). All participating institute's Institutional Review Boards approved the studies and the procedures and all participants provided written informed consent before study participation. The rh-IL-7 study (referred to as Study I) [28] evaluated Recombinant Human Interleukin 7 (rh-IL-7), a nonglycosylated protein composed of 153 amino acids, and included 14 HIV-infected patients receiving antiretroviral therapy whose CD4^+^ T cell counts were between 100 and 400 cells/µl and whose plasma HIV RNA levels were less than 50 copies/ml. Patients received a total of 8 subcutaneous injections of 2 different doses of recombinant human IL-7 (3 or 10 µg/kg, dose 1 and 2, respectively) 3 times per week over a 16-day period. Eleven repeated measurements of total CD4^+^ T cells up to 48 weeks and four measurements of Ki67^+^ positive T cells among CD4^+^ T cells up to 12 weeks were performed.

The INSPIRE Study (referred to as Study II) [30] evaluated 3 weekly subcutaneous (SC) injections of a purified glycosylated 152 amino acid rhIL-7 (CYT107 over a period of 2 weeks). Three doses were tested: 10, 20 and 30 µg/Kg/week. Seven, 8 and 6 patients received three injections (one per week) in each dose group, respectively. Two HIV-infected patients were randomized per dose level and received a placebo (NaCl). Visits for safety and immunologic evaluation were performed at days 7, 14, 21, 28, 35, week 9 and week 12 and then quarterly up to week 52. In the INSPIRE 2 Study (referred to as Study III), 12 patients received 3 subcutaneous injection of 20 µg/Kg/week CYT107 (one per week over 2 weeks) and were followed up to week 52.

### Flow cytometry

Absolute CD4 T-cell counts, T cell expression of Ki67, and the proportions of naive subsets defined by expression of CD45RA and CD27 (naive: CD45RA^+^CD27^+^) were measured in whole blood by flow cytometric assays within 6 hours of blood draw in the Rh-IL7 study [28]. In INSPIRE, naive cells in cryopreserved samples were identified by expression of CD45RA, CD27 and CCR7; in INSPIRE 2 naive cells were enumerated in cryopreserved PBMC by expression of CD45RA and CCR7.

### Mathematical model for the estimation of IL-7 effect

Ki67 is a cellular marker of proliferation [56] and is associated with cell proliferation. It is present during all active phases of the cell cycle (namely G_1_, S, G_2_ and M) and it is absent from resting cells (phase G_0_). Therefore, some of the cells expressing Ki67 are actually in the division phase M and the rest are “on their way” to this phase or very recently in this phase. In this model, we assume that proliferating cells express Ki67 whereas non-proliferating (i.e. resting) cells do not; this is a relatively good approximation. We consider the following mathematical model including two populations of cells (see Figure S2 for a general cartoon of the model): non-proliferating cells (Ki67^−^, denoted Q) and proliferating cells (Ki67^+^, denoted P):
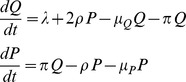



Non-proliferating cells (Q) are produced at a constant rate λ. They become Ki67^+^ at rate π and die at rate μ_Q_. Proliferating cells (P) die at rate μ_p_ and lose their proliferation marker Ki67 at rate ρ (cells express Ki67 for 1/ρ days). We assumed that 2*ρP* cells enter the Q compartment that is two daughter cells are produced after one single cell cycle. The loss rates (μ_P_ and μ_Q_) are influenced by cell survival but also by any redistribution between blood and other tissues. Before the first injection at *t* = 0, both populations are assumed to be at equilibrium (i.e. 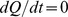 and 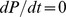). This model was used for total CD4^+^ T-cells where Q and P include both naive and memory CD4^+^ T-cells. Similarly, we used this general model to describe naive CD4^+^ T-cell dynamics adding a superscript ‘N’ to all parameters (λ^N^, π^N^, μ_Q_^N^, μ_P_^N^, ρ^N^) and where Q^N^ represent non-proliferating naive CD4^+^ T-cells and P^N^ proliferating naive CD4^+^ T-cells.

To model the effect of IL-7, we considered different models allowing some parameters to change over time (i.e. during or after the IL-7 administration). We distinguished three models:

Model 1 includes only an effect on the proliferation rate π during the IL-7 administration

Model 2 includes an effect on π and an additional effect on the loss rate of non-proliferation cells μ_Q_ (effect tested during or after the IL-7 administration)

Model 3 includes an effect on π and an additional effect on the production rate λ (effect tested during or after the IL-7 administration).

Models including an effect of IL-7 on the duration of Ki67 expression or on a direct production of Ki67^+^ cells from the thymus were also tried but did not improve the fit of the data (not shown).

Each parameter is assumed equal to a baseline value (denoted π_0_, μ_Q0_, λ_0_) and we tested a possible effect of IL-7 and a possible dose effect. For instance, the proliferation rate π was assumed equal to 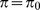 before (*t* = 0) and after IL-7 administration (*t*>τ) and to 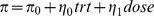 during IL-7 administration (0<*t*≤*τ*). The variable *trt* indicates if an individual received the placebo (*trt* = 0) or IL-7 injections (*trt* = 1), hence the parameter η_0_ represents the possible effect of IL-7 on the parameter π. Similarly, we tested a possible dose effect via the parameter η_1_ and the variable *dose*; *dose* = 0 if the individual received the placebo and 0.3, 1, 2 or 3 according to the received dose of IL-7. The additional dose effect is here assumed to be linear: the dose 30 µg/Kg will have 3 times more effect than the dose 10 µg/Kg and 10 times more than the dose 3 µg/Kg. The time τ (time of IL-7 effect on the proliferation rate π) was fixed to 16 days and robustness analyses with values between 14 and 16 days have been performed leading to similar results but slightly worse likelihood (not shown).

### Mathematical model of successive IL-7 injections

For the simulations of repeated administrations of IL-7, a homeostatic control of proliferation was incorporated in order to constrain the CD4^+^ T cell level in a credible range (below 1500 cells/µL). The newly defined rate of proliferation, denoted π*, is defined as follows:
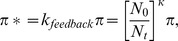
where *N*_0_ is the baseline CD4 T cell count, *N_t_* is CD4 T cell count at time *t* and *κ* is a number lower than 1 estimated on the data. As *N*_0_ is relatively low due to lymphopenia, cells will be allowed to proliferate more while the circulating number of CD4+ T cells is still relatively low but as soon as this number deviates too much from the baseline value the proliferation rate is reduced [57]. Several formulations have been tested, including one with N_max_ = 1000 referring to a normal healthy value rather than N_0_ and all formulations led to similar conclusions due to different estimates of the parameter *κ* (not shown). The parameter *κ* was estimated by profile likelihood because it was difficult to estimate it at the same time as the other parameters. The parameter *κ* was therefore fixed at different plausible values and the model was estimated for each value of *κ* and we kept the value of *κ* for which the model had the lowest likelihood.

Here, we only considered Model 2 including the effect of IL-7 on the proliferation rate and the loss rate of non-proliferating cells as it was the best model according to real data. Moreover, in this model, the loss rate of non-proliferating cell (μ_Q_) was assumed decreased (to a constant value) after day 16 and during (T_full_-16) days after the injection and then linearly increased to its baseline value after T_end_ days (see Figure S2). As subsequent cycles of IL-7 might have reduced effects compared to the first one, the proliferation rate (resp. loss rate) of the subsequent cycle is denoted by π_sub_ (resp. μ_Qsub_) and keep π (resp. μ_Q_) for the first injection. These rates are defined as π_sub_ = α_π_π and μ_Qsub_ = α_μQ_μ_Q_, where α_π_ and α_μQ_ represent the strength of new IL-7 administration (i.e. the percentage of effect of π_sub_ and μ_Qsub_ compared to the initial injection). Therefore, we assumed for all subsequent cycles a similar reduction of IL-7 effects compared to the first cycle.

### Statistical methods

Parameters were estimated using maximum penalized likelihood that takes into account unbalanced data due to sparse missing values and the availability of Ki67^+^ staining up to week 12 (no measurements were available beyond that time). This method can be viewed either as a version of maximum likelihood allowing taking into account prior knowledge (from previous estimates found in published studies), or as an approximation of Bayesian inference [58], [59].

The Ordinary Differential Equations (ODE) system was solved with dsolve from the ODEPACK [60] for stiff system using Backward Differentiation Formula (BDF) methods (the Gear methods). Each parameter (θ_i_) was modeled as the sum of a population (fixed) parameter (β) and a random effect (b_i_) allowing the parameter to be different from one patient to another: 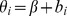 Each random effect was assumed to be normally distributed with a variance to be estimated: 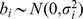. A stepwise selection procedure was used and when the variance of a given random effect was not significant, the parameter was considered as fixed in the next model. We observe the number of proliferating cells (*P*) and the total number of cells (*P+Q*) plus a measurement error adding two unknown parameters 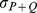 and 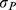. The final models included only two random effects, one for λ (production rate) and one for ρ (the rate at which proliferating cells go back to rest). The best model was selected using an approximation of the likelihood cross-validation criterion (LCVa, [58], [59]) that is based on the likelihood weighted by the number of parameters estimated like the Akaike Criteria (AIC). The lower is the value of the criteria the better is the model. Individual predicted trajectories were computed using the Parametric Empirical Bayes (PEB) for all parameters having a random effect [61].

## Supporting Information

Figure S1 **Dose-dependent increase of total CD4^+^ T cell count (A), Ki67+CD4+ T cells count (B) and percentage of CD4+ T cells expressing Ki67 (C) for Study rh-IL7 (Study I).** Observed median count in cells/µL by group: 3 µg/kg (black dots) and 10 µg/kg (grey dots). Error bars and other statistical analyses are provided in Levy et al. (2009).
(DOC)

Figure S2 **Graphical representation of the biological model (A) and changes of the loss rate over time after the first IL-7 cycle (B).** After the first cycle, the potential effect of IL-7 might be reduced and results in a higher level of μ_Q_ between day 16 and day T_full_. In the simulation study, we considered two sets of values for T_full_ and T_end_, namely (T_full_ = 90, T_end_ = 365) and (T_full_ = 270 and T_end_ = 731).
(DOC)

Figure S3 **Goodness of fit of total CD4^+^ T cell count from rh-IL-7 Study (Study I) for the three different models.** The prediction from model 1 assuming only an effect of IL-7 on the proliferation rates is in solid line, from Model 2 assuming an effect on proliferation rate and on the loss rate of resting cells in dashed line and from Model 3 assuming an effect on proliferation rate and on the thymic production in dotted lines. Note that the estimated trajectories from Model 2 and 3 almost overlap.
(DOC)

Figure S4 **Goodness of fit of total CD4^+^ T cell count from INSPIRE Study (Study II) for the three different models.** The prediction from model 1 assuming only an effect of IL-7 on the proliferation rates is in solid line, from Model 2 assuming an effect on proliferation rate and on the loss rate of resting cells in dashed line and from Model 3 assuming an effect on proliferation rate and on the thymic production in dotted lines. Note that the estimated trajectories from Model 2 and 3 almost overlap.
(DOC)

Figure S5 **Goodness of fit of naive CD4^+^ T cell count from INSPIRE Study (Study II) for the three different models.** The prediction from model 1 assuming only an effect of IL-7 on the proliferation rates is in solid line, from Model 2 assuming an effect on proliferation rate and on the loss rate of resting cells in dashed line and from Model 3 assuming an effect on proliferation rate and on the thymic production in dotted lines. Note that the estimated trajectories from Model 2 and 3 almost overlap.
(DOC)

Figure S6 **Predicted dynamics of total CD4+ T cell count for the 9 first patients from Study III (INSPIRE 2).** Dynamics were predicted using Model 2, assuming an effect of IL-7 on proliferation and loss rate of non-proliferating cells after the IL-7 administration. The first two measurements (at the left side of the vertical line) were used to compute the individual parametric empirical bayes. The dynamics at the right side of the vertical line were predicted without using measurements subsequent to the first two. 95% measurement error confidence intervals are represented by dashed lines.
(DOC)

Figure S7 **Median percentages of time spent above 500 cells/µL and median numbers of cycles over a 24 month follow-up.** For the simulations of repeated administrations of IL-7, we assumed that injections might have reduced effects compared to the first one of (1-α_π_)% and (1-α_μQ_)%.
(DOC)

Table S1 **Estimates of model parameters for total CD4^+^ and CD4^+^Ki67^+^ T-cell dynamics in Study II (INSPIRE Study).** Model 1: only the proliferation rate (π) is modified; Model 2: proliferation rate (π) and loss rate (μ_Q_) of non-proliferating cells are modified; Model 3: proliferation rate and constant production rate (λ) are modified. All IL-7 effects underlined in grey were statistically significant at 0.05 level. Standard-errors are given between brackets.
(DOC)

Table S2 **Estimates of model parameters for total CD4^+^ and CD4^+^Ki67^+^ T-cell dynamics in Study I (rh-IL-7 study).** Model 1: only the proliferation rate (π) is modified; Model 2: proliferation rate (π) and loss rate (μ_Q_) of non-proliferating cells are modified; Model 3: proliferation rate and constant production rate (λ) are modified. All IL-7 effects underlined in grey were statistically significant at 0.05 level. Standard-errors are given between brackets.
(DOC)

Table S3 **Estimates of model parameters for naive CD4^+^ and naive CD4^+^Ki67^+^ T-cell dynamics in Study II (INSPIRE Study).** Model 1: only the proliferation rate (π^N^) is modified; Model 2: proliferation rate (π^N^) and loss rate (μ_Q_^N^) of non-proliferating cells are modified; Model 3: proliferation rate and constant production rate (λ^N^) are modified. All IL-7 effects underlined in grey were statistically significant at 0.05 level. Standard-errors are given between brackets.
(DOC)

Table S4 **Percentage of time spent above 500 cells/µL, number and median time between cycles according to various scenarios.**
(DOC)

## References

[1] Lange CG, Lederman MM (2003) Immune reconstitution with antiretroviral therapies in chronic HIV-1 infection. Journal of Antimicrobial Chemotherapy 51: 1–4. 12493779 10.1093/jac/dkg071

[2] Fry TJ, Mackall CL (2002) Interleukin-7: from bench to clinic. Blood 99: 3892–3904. 12010786 10.1182/blood.v99.11.3892

[3] Mackall CL, Fry TJ, Gress RE (2011) Harnessing the biology of IL-7 for therapeutic application. Nature Reviews Immunology 11: 330–342. 10.1038/nri2970 PMC7351348 21508983

[4] Mackall CL, Fry TJ, Bare C, Morgan P, Galbraith A, et al (2001) IL-7 increases both thymic-dependent and thymic-independent T-cell regeneration after bone marrow transplantation. Blood 97: 1491–1497. 11222398 10.1182/blood.v97.5.1491

[5] Bolotin E, Smogorzewska M, Smith S, Widmer M, Weinberg K (1996) Enhancement of thymopoiesis after bone marrow transplant by in vivo interleukin-7. Blood 88: 1887–1894. 8781449

[6] Okamoto Y, Douek DC, McFarland RD, Koup RA (2002) Effects of exogenous interleukin-7 on human thymus function. Blood 99: 2851–2858. 11929775 10.1182/blood.v99.8.2851

[7] Soares MV, Borthwick NJ, Maini MK, Janossy G, Salmon M, et al (1998) IL-7-dependent extrathymic expansion of CD45RA+ T cells enables preservation of a naive repertoire. Journal of Immunology 161: 5909–5917. 9834071

[8] Swainson L, Kinet S, Mongellaz C, Sourisseau M, Henriques T, et al (2007) IL-7-induced proliferation of recent thymic emigrants requires activation of the PI3K pathway. Blood 109: 1034–1042. 17023582 10.1182/blood-2006-06-027912

[9] Sportes C, Hakim FT, Memon SA, Zhang H, Chua KS, et al (2008) Administration of rhIL-7 in humans increases in vivo TCR repertoire diversity by preferential expansion of naive T cell subsets. Journal of Experimental Medicine 205: 1701–1714. 18573906 10.1084/jem.20071681 PMC2442646

[10] Fry TJ, Christensen BL, Komschlies KL, Gress RE, Mackall CL (2001) Interleukin-7 restores immunity in athymic T-cell-depleted hosts. Blood 97: 1525–1533. 11238086 10.1182/blood.v97.6.1525

[11] Tan JT, Dudl E, LeRoy E, Murray R, Sprent J, et al (2001) IL-7 is critical for homeostatic proliferation and survival of naive T cells. Proceedings of the National Academy of Sciences of the United States of America 98: 8732–8737. 11447288 10.1073/pnas.161126098 PMC37504

[12] Vella AT, Dow S, Potter TA, Kappler J, Marrack P (1998) Cytokine-induced survival of activated T cells in vitro and in vivo. Proceedings of the National Academy of Sciences of the United States of America 95: 3810–3815. 9520449 10.1073/pnas.95.7.3810 PMC19919

[13] Seddon B, Tomlinson P, Zamoyska R (2003) Interleukin 7 and T cell receptor signals regulate homeostasis of CD4 memory cells. Nature Immunology 4: 680–686. 12808452 10.1038/ni946

[14] Kondrack RM, Harbertson J, Tan JT, McBreen ME, Surh CD, et al (2003) Interleukin 7 regulates the survival and generation of memory CD4 cells. Journal of Experimental Medicine 198: 1797–1806. 14662907 10.1084/jem.20030735 PMC2194153

[15] Leone A, Rohankhedkar M, Okoye A, Legasse A, Axthelm MK, et al (2010) Increased CD4+ T cell levels during IL-7 administration of antiretroviral therapy-treated simian immunodeficiency virus-positive macaques are not dependent on strong proliferative responses. Journal of Immunology 185: 1650–1659. 10.4049/jimmunol.0902626 PMC3050806 20622118

[16] Fry TJ, Connick E, Falloon J, Lederman MM, Liewehr DJ, et al (2001) A potential role for interleukin-7 in T-cell homeostasis. Blood 97: 2983–2990. 11342421 10.1182/blood.v97.10.2983

[17] Mastroianni CM, Forcina G, d'Ettorre G, Lichtner M, Mengoni F, et al (2001) Circulating levels of interleukin-7 in antiretroviral-naive and highly active antiretroviral therapy-treated HIV-infected patients. HIV Clinical Trials 2: 108–112. 11590518 10.1310/6V29-4UU5-Y3FP-JERT

[18] Beq S, Delfraissy JF, Theze J (2004) Interleukin-7 (IL-7): immune function, involvement in the pathogenesis of HIV infection and therapeutic potential. European Cytokine Network 15: 279–289. 15627636

[19] Rajasuriar R, Booth D, Solomon A, Chua K, Spelman T, et al (2010) Biological determinants of immune reconstitution in HIV-infected patients receiving antiretroviral therapy: the role of interleukin 7 and interleukin 7 receptor alpha and microbial translocation. The Journal of Infectious Diseases 202: 1254–1264. 20812848 10.1086/656369

[20] Hodge JN, Srinivasula S, Hu Z, Read SW, Porter BO, et al (2011) Decreases in IL-7 levels during antiretroviral treatment of HIV infection suggest a primary mechanism of receptor-mediated clearance. Blood 118: 3244–3253. 21778338 10.1182/blood-2010-12-323600 PMC3179394

[21] Guimond M, Veenstra RG, Grindler DJ, Zhang H, Cui Y, et al (2009) Interleukin 7 signaling in dendritic cells regulates the homeostatic proliferation and niche size of CD4+ T cells. Nature Immunology 10: 149–157. 19136960 10.1038/ni.1695 PMC2713006

[22] Shive CL, Mudd JC, Funderburg NT, Sieg SF, Kyi B, et al (2014) Inflammatory Cytokines Drive CD4 T cell Cycling and Impaired Responsiveness to Interleukin-7: Implications for Immune Failure in HIV Disease. Journal of Infectious Diseases [Epub ahead of print]: doi: 10.1093/infdis/jiu125 10.1093/infdis/jiu125 PMC4172041 24585897

[23] Zeng M, Smith AJ, Wietgrefe SW, Southern PJ, Schacker TW, et al (2011) Cumulative mechanisms of lymphoid tissue fibrosis and T cell depletion in HIV-1 and SIV infections. Journal of Clinical Investigation 121: 998–1008. 21393864 10.1172/JCI45157 PMC3049394

[24] Juffroy O, Bugault F, Lambotte O, Landires I, Viard JP, et al (2010) Dual mechanism of impairment of interleukin-7 (IL-7) responses in human immunodeficiency virus infection: decreased IL-7 binding and abnormal activation of the JAK/STAT5 pathway. Journal of Virology 84: 96–108. 19864382 10.1128/JVI.01475-09 PMC2798395

[25] Beq S, Nugeyre MT, Ho Tsong Fang R, Gautier D, Legrand R, et al (2006) IL-7 induces immunological improvement in SIV-infected rhesus macaques under antiviral therapy. Journal of Immunology 176: 914–922. 10.4049/jimmunol.176.2.914 16393976

[26] Fry TJ, Moniuszko M, Creekmore S, Donohue SJ, Douek DC, et al (2003) IL-7 therapy dramatically alters peripheral T-cell homeostasis in normal and SIV-infected nonhuman primates. Blood 101: 2294–2299. 12411295 10.1182/blood-2002-07-2297

[27] Sereti I, Dunham RM, Spritzler J, Aga E, Proschan MA, et al (2009) IL-7 administration drives T cell-cycle entry and expansion in HIV-1 infection. Blood 113: 6304–6314. 19380868 10.1182/blood-2008-10-186601 PMC2710926

[28] Levy Y, Lacabaratz C, Weiss L, Viard JP, Goujard C, et al (2009) Enhanced T cell recovery in HIV-1-infected adults through IL-7 treatment. Journal of Clinical Investigation 119: 997–1007. 19287090 10.1172/JCI38052 PMC2662568

[29] Camargo JF, Kulkarni H, Agan BK, Gaitan AA, Beachy LA, et al (2009) Responsiveness of T cells to interleukin-7 is associated with higher CD4+ T cell counts in HIV-1-positive individuals with highly active antiretroviral therapy-induced viral load suppression. The Journal of Infectious Diseases 199: 1872–1882. 19432535 10.1086/598858 PMC3777824

[30] Levy Y, Sereti I, Tambussi G, Routy J-P, Lelievre JD, et al (2012) Effects of Recombinant Human Interleukin 7 on T Cell Recovery and Thymic Output in HIV-infected Patients receiving antiretroviral therapy: results of a Phase I/IIa Randomized, Placebo Controlled, Multicenter Study. Clinical Infectious Diseases 55: 291–300. 22550117 10.1093/cid/cis383 PMC3381639

[31] Douek DC, McFarland RD, Keiser PH, Gage EA, Massey JM, et al (1998) Changes in thymic function with age and during the treatment of HIV infection. Nature 396: 690–695. 9872319 10.1038/25374

[32] Bains I, Thiebaut R, Yates AJ, Callard R (2009) Quantifying thymic export: combining models of naive T cell proliferation and TCR excision circle dynamics gives an explicit measure of thymic output. Journal of Immunology 183: 4329–4336. 10.4049/jimmunol.0900743 19734223

[33] Ribeiro RM, Mohri H, Ho DD, Perelson AS (2002) In vivo dynamics of T cell activation, proliferation, and death in HIV-1 infection: Why are CD4(+) but not CD8(+) T cells depleted? Proceedings of the National Academy of Sciences of the United States of America 99: 15572–15577. 12434018 10.1073/pnas.242358099 PMC137758

[34] Hellerstein M, Hanley MB, Cesar D, Siler S, Papageorgopoulos C, et al (1999) Directly measured kinetics of circulating T lymphocytes in normal and HIV-1-infected humans. Nature Medicine 5: 83–89. 10.1038/4772 9883844

[35] Kovacs JA, Lempicki RA, Sidorov IA, Adelsberger JW, Herpin B, et al (2001) Identification of dynamically distinct subpopulations of T lymphocytes that are differentially affected by HIV. Journal of Experimental Medicine 194: 1731–1741. 11748275 10.1084/jem.194.12.1731 PMC2193579

[36] Bains I, Antia R, Callard R, Yates AJ (2009) Quantifying the development of the peripheral naive CD4+ T-cell pool in humans. Blood 113: 5480–5487. 19179300 10.1182/blood-2008-10-184184 PMC2689049

[37] den Braber I, Mugwagwa T, Vrisekoop N, Westera L, Mogling R, et al (2012) Maintenance of peripheral naive T cells is sustained by thymus output in mice but not humans. Immunity 36: 288–297. 22365666 10.1016/j.immuni.2012.02.006

[38] Abrams D, Levy Y, Losso MH, Babiker A, Collins G, et al (2009) Interleukin-2 therapy in patients with HIV infection. The New England Journal of Medicine 361: 1548–1559. 19828532 10.1056/NEJMoa0903175 PMC2869083

[39] Weiss L, Letimier FA, Carriere M, Maiella S, Donkova-Petrini V, et al (2010) In vivo expansion of naive and activated CD4+CD25+FOXP3+ regulatory T cell populations in interleukin-2-treated HIV patients. Proceedings of the National Academy of Sciences of the United States of America 107: 10632–10637. 20498045 10.1073/pnas.1000027107 PMC2890853

[40] Porter BO, Shen J, Kovacs JA, Davey RT, Rehm C, et al (2009) Interleukin-2 cycling causes transient increases in high-sensitivity C-reactive protein and D-dimer that are not associated with plasma HIV-RNA levels. AIDS 23: 2015–2019. 19617815 10.1097/QAD.0b013e32832d72c6 PMC2760947

[41] Reynolds J, Coles M, Lythe G, Molina-Paris C (2013) Mathematical Model of Naive T Cell Division and Survival IL-7 Thresholds. Frontiers in Immunology 4: 434. 24391638 10.3389/fimmu.2013.00434 PMC3870322

[42] Vivien L, Benoist C, Mathis D (2001) T lymphocytes need IL-7 but not IL-4 or IL-6 to survive in vivo. International immunology 13: 763–768. 11369703 10.1093/intimm/13.6.763

[43] Storek J, Gillespy T 3rd, Lu H, Joseph A, Dawson MA, et al (2003) Interleukin-7 improves CD4 T-cell reconstitution after autologous CD34 cell transplantation in monkeys. Blood 101: 4209–4218. 12543864 10.1182/blood-2002-08-2671

[44] Park JH, Yu Q, Erman B, Appelbaum JS, Montoya-Durango D, et al (2004) Suppression of IL7Ralpha transcription by IL-7 and other prosurvival cytokines: a novel mechanism for maximizing IL-7-dependent T cell survival. Immunity 21: 289–302. 15308108 10.1016/j.immuni.2004.07.016

[45] Mazzucchelli R, Durum SK (2007) Interleukin-7 receptor expression: intelligent design. Nature Reviews in Immunology 7: 144–154. 10.1038/nri2023 17259970

[46] Sereti I, Estes JD, Thompson WL, Morcock DR, Fischl MA, et al (2014) Decreases in colonic and systemic inflammation in chronic HIV infection after IL-7 administration. PLoS Pathogens 10: e1003890. 24497828 10.1371/journal.ppat.1003890 PMC3907377

[47] Huang M, Sharma S, Zhu LX, Keane MP, Luo J, et al (2002) IL-7 inhibits fibroblast TGF-beta production and signaling in pulmonary fibrosis. The Journal of Clinical Investigation 109: 931–937. 11927620 10.1172/JCI14685 PMC150933

[48] Meier D, Bornmann C, Chappaz S, Schmutz S, Otten LA, et al (2007) Ectopic lymphoid-organ development occurs through interleukin 7-mediated enhanced survival of lymphoid-tissue-inducer cells. Immunity 26: 643–654. 17521585 10.1016/j.immuni.2007.04.009

[49] Onder L, Narang P, Scandella E, Chai Q, Iolyeva M, et al (2012) IL-7-producing stromal cells are critical for lymph node remodeling. Blood 120: 4675–4683. 22955921 10.1182/blood-2012-03-416859 PMC3952724

[50] Beq S, Rozlan S, Gautier D, Parker R, Mersseman V, et al (2009) Injection of glycosylated recombinant simian IL-7 provokes rapid and massive T-cell homing in rhesus macaques. Blood 114: 816–825. 19351957 10.1182/blood-2008-11-191288

[51] Li J, Huston G, Swain SL (2003) IL-7 promotes the transition of CD4 effectors to persistent memory cells. Journal of Experimental Medicine 198: 1807–1815. 14676295 10.1084/jem.20030725 PMC2194161

[52] Chomont N, El-Far M, Ancuta P, Trautmann L, Procopio FA, et al (2009) HIV reservoir size and persistence are driven by T cell survival and homeostatic proliferation. Nature Medicine 15: 893–U892. 10.1038/nm.1972 PMC2859814 19543283

[53] Prague M, Commenges D, Thiebaut R (2013) Dynamical models of biomarkers and clinical progression for personalized medicine: The HIV context. Advanced Drug Delivery Review 65: 954–965. 10.1016/j.addr.2013.04.004 23603207

[54] Westera L, Zhang Y, Tesselaar K, Borghans JA, Macallan DC (2013) Quantitating lymphocyte homeostasis in vivo in humans using stable isotope tracers. Methods in Molecular Biology 979: 107–131. 23397392 10.1007/978-1-62703-290-2_10

[55] Kovacs JA, Lempicki RA, Sidorov IA, Adelsberger JW, Sereti I, et al (2005) Induction of prolonged survival of CD4(+) T lymphocytes by intermittent IL-2 therapy in HIV-infected patients. Journal of Clinical Investigation 115: 2139–2148. 16025158 10.1172/JCI23196 PMC1174914

[56] Scholzen T, Gerdes J (2000) The Ki-67 protein: from the known and the unknown. Journal of Cell Physiology 182: 311–322. 10.1002/(SICI)1097-4652(200003)182:3<311::AID-JCP1>3.0.CO;2-9 10653597

[57] Rosenbaum SE (2011). Basic pharmacokinetics and pharmacodynamics: an integrated textbook and computer simulations. First edition ed: Wiley & Sons, Hoboken New Jersey. pp. 352–356.

[58] Drylewicz J, Commenges D, Thiébaut R (2012) on behalf of the CASCADE Collaboration (2012) Maximum a Posteriori estimation in dynamical models of primary HIV infection. Statistical Communications in Infectious Diseases 4 DOI: 10.1515/1948-4690.1040

[59] Prague M, Commenges D, Guedj J, Drylewicz J, Thiébaut R (2013) NIMROD: A Program for Inference via Normal Approximation of the Posterior in Models with Random effects based on Ordinary Differential Equations. Computer Methods and Programs in Biomedicine 111: 447–458. 23764196 10.1016/j.cmpb.2013.04.014

[60] Hindmarsh AC (1983) ODEPACK, A Systematized Collection of ODE Solvers. IMACS Transactions on Scientific Computation 1: 55–64.

[61] Kass RE, Steffey D (1989) Approximate Bayesian inference in conditionally independent hierarchical models (parametric empirical Bayes models). Journal of the American Statistical Association 84: 717–726.

