# Supplementary material for: Quantifying and Predicting the Effect of Exogenous Interleukin-7 on CD4+T Cells in HIV-1 Infection
Source: PLoS Comput Biol. 2014 May 22;10(5):e1003630. doi: 10.1371/journal.pcbi.1003630 (PMC4031052; doi:10.1371/journal.pcbi.1003630)
Supplement: Figure S1 — Dose-dependent increase of total CD4+ T cell count (A), Ki67+CD4+ T cells count (B) and percentage of CD4+ T cells expressing Ki67 (C) for Study rh-IL7 (Study I). Observed median count in cells/µL by group: 3 µg/kg (black dots) and 10 µg/kg (grey dots). Error bars and other statistical analyses are provided in Levy et al. (2009). (DOC) [file pcbi.1003630.s001.doc]

**
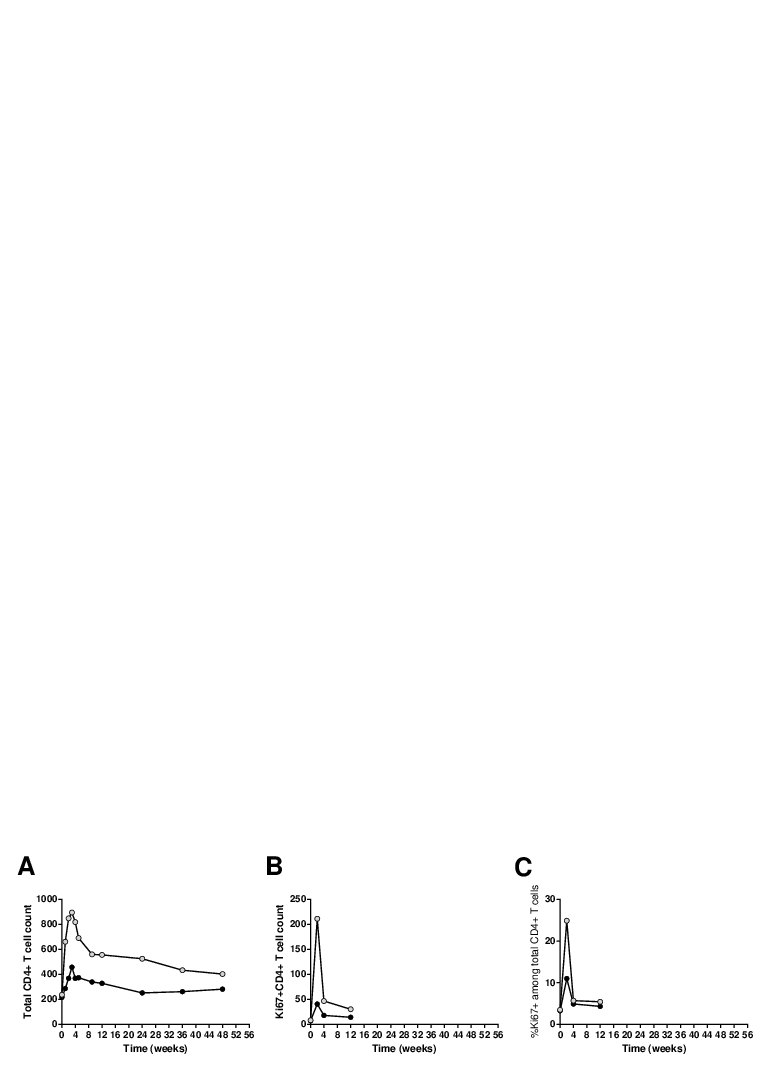
**

**Figure S1. Dose-dependent increase of total CD4^+^ T cell count (A) , Ki67+CD4+ T cells count (B) and percentage of CD4+ T cells expressing Ki67 (C) for Study rh-IL7 (Study I).** Observed median count in cells/µL by group: 3µg/kg (black dots) and 10µg/kg (grey dots). Error bars and other statistical analyses are provided in Levy et al. (2009)
