# Supplementary material for: Quantifying and Predicting the Effect of Exogenous Interleukin-7 on CD4+T Cells in HIV-1 Infection
Source: PLoS Comput Biol. 2014 May 22;10(5):e1003630. doi: 10.1371/journal.pcbi.1003630 (PMC4031052; doi:10.1371/journal.pcbi.1003630)
Supplement: Figure S2 — Graphical representation of the biological model (A) and changes of the loss rate over time after the first IL-7 cycle (B). After the first cycle, the potential effect of IL-7 might be reduced and results in a higher level of μQ between day 16 and day Tfull. In the simulation study, we considered two sets of values for Tfull and Tend, namely (Tfull = 90, Tend = 365) and (Tfull = 270 and Tend = 731). (DOC) [file pcbi.1003630.s002.doc]

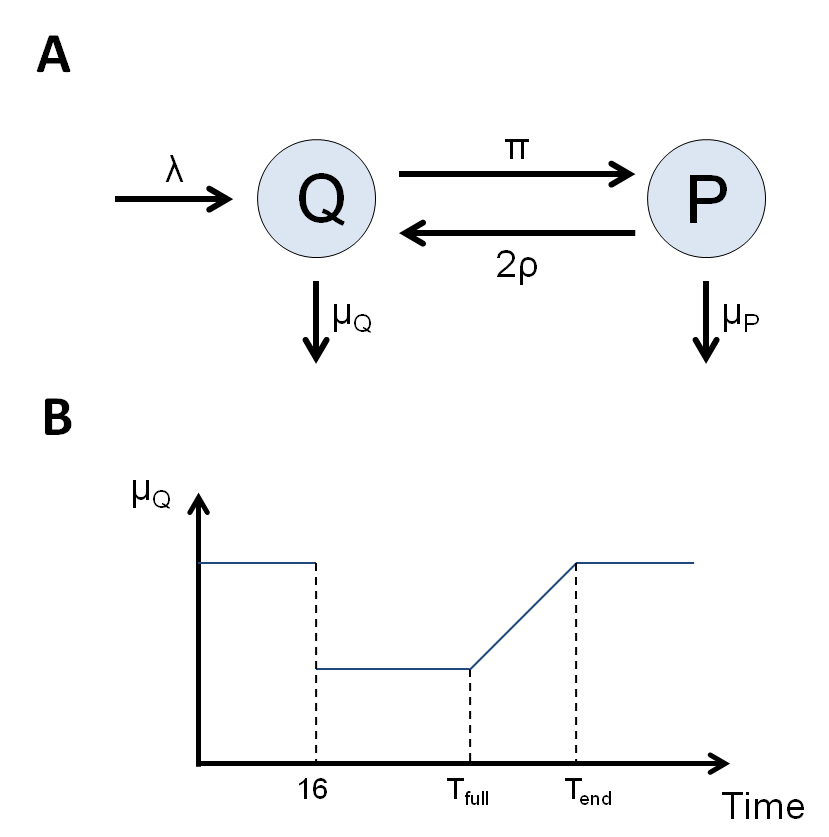


**Figure S2. Graphical representation of the biological model (A) and changes of the loss rate over time after the first IL-7 cycle (B).** After the first cycle, the potential effect of IL-7 might be reduced and results in a higher level of μ_Q_ between day 16 and day T_full_. In the simulation study, we considered two sets of values for T_full_ and T_end_, namely (T_full_=90, T_end_=365) and (T_full_=270 and T_end_=731).
