# Supplementary material for: Quantifying and Predicting the Effect of Exogenous Interleukin-7 on CD4+T Cells in HIV-1 Infection
Source: PLoS Comput Biol. 2014 May 22;10(5):e1003630. doi: 10.1371/journal.pcbi.1003630 (PMC4031052; doi:10.1371/journal.pcbi.1003630)
Supplement: Figure S5 — Goodness of fit of naive CD4+ T cell count from INSPIRE Study (Study II) for the three different models. The prediction from model 1 assuming only an effect of IL-7 on the proliferation rates is in solid line, from Model 2 assuming an effect on proliferation rate and on the loss rate of resting cells in dashed line and from Model 3 assuming an effect on proliferation rate and on the thymic production in dotted lines. Note that the estimated trajectories from Model 2 and 3 almost overlap. (DOC) [file pcbi.1003630.s005.doc]

**Figure S5. Goodness of fit of naive CD4^+^ T cell count from INSPIRE Study (Study II) for the three different models.** The prediction from model 1 assuming only an effect of IL-7 on the proliferation rates is in solid line, from Model 2 assuming an effect on proliferation rate and on the loss rate of resting cells in dashed line and from Model 3 assuming an effect on proliferation rate and on the thymic production in dotted lines. Note that the estimated trajectories from Model 2 and 3 almost overlap.
