# Supplementary material for: Quantifying and Predicting the Effect of Exogenous Interleukin-7 on CD4+T Cells in HIV-1 Infection
Source: PLoS Comput Biol. 2014 May 22;10(5):e1003630. doi: 10.1371/journal.pcbi.1003630 (PMC4031052; doi:10.1371/journal.pcbi.1003630)
Supplement: Figure S6 — Predicted dynamics of total CD4+ T cell count for the 9 first patients from Study III (INSPIRE 2). Dynamics were predicted using Model 2, assuming an effect of IL-7 on proliferation and loss rate of non-proliferating cells after the IL-7 administration. The first two measurements (at the left side of the vertical line) were used to compute the individual parametric empirical bayes. The dynamics at the right side of the vertical line were predicted without using measurements subsequent to the first two. 95% measurement error confidence intervals are represented by dashed lines. (DOC) [file pcbi.1003630.s006.doc]

**
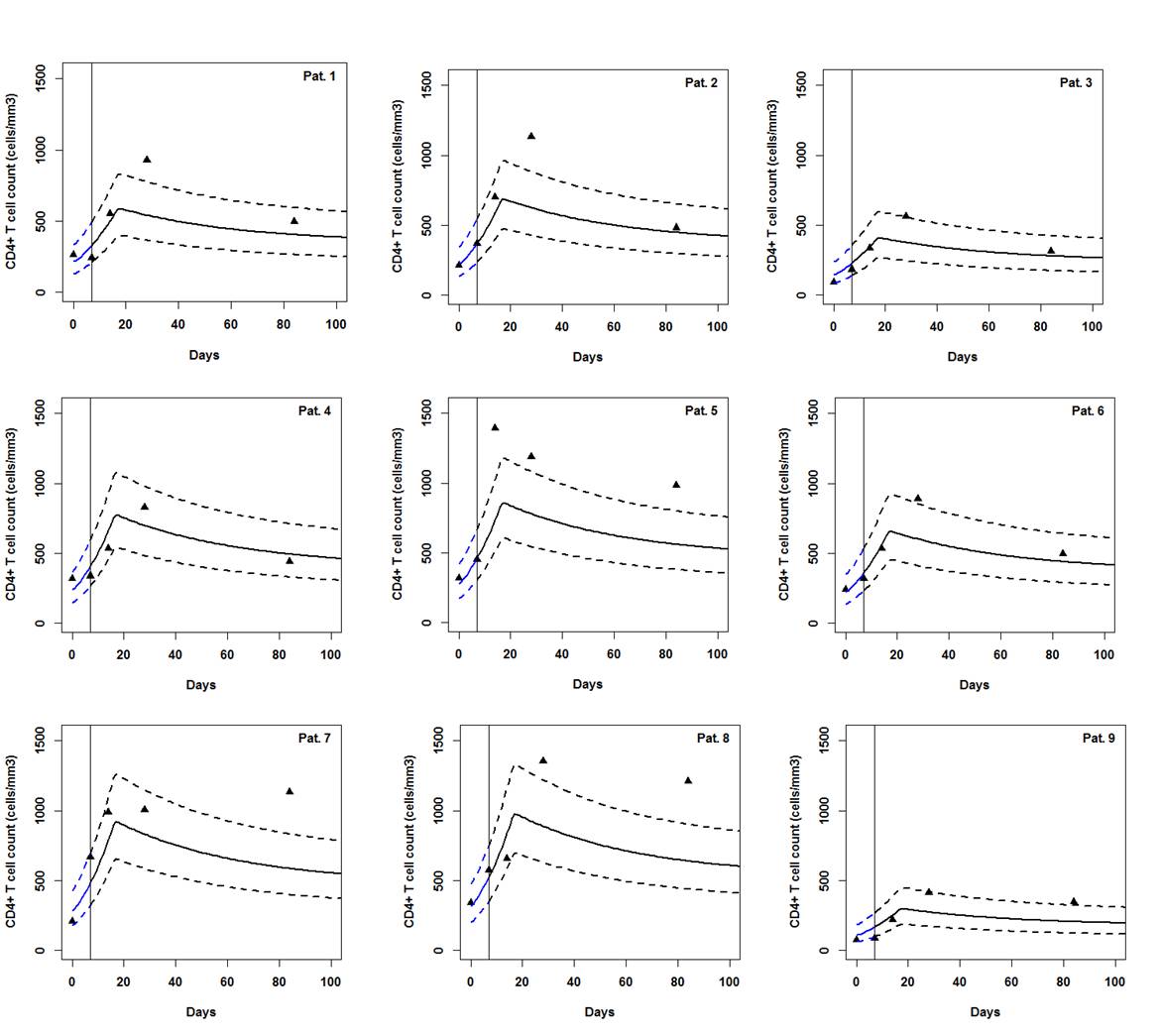
**

**Figure S6. Predicted dynamics of total CD4+ T cell count for the 9 first patients from Study III (INSPIRE 2).** Dynamics were predicted using Model 2, assuming an effect of IL-7 on proliferation and loss rate of non-proliferating cells after the IL-7 administration. The first two measurements (at the left side of the vertical line) were used to compute the individual parametric empirical bayes. The dynamics at the right side of the vertical line were predicted without using measurements subsequent to the first two. 95% measurement error confidence intervals are represented by dashed lines.
