# Supplementary material for: Quantifying and Predicting the Effect of Exogenous Interleukin-7 on CD4+T Cells in HIV-1 Infection
Source: PLoS Comput Biol. 2014 May 22;10(5):e1003630. doi: 10.1371/journal.pcbi.1003630 (PMC4031052; doi:10.1371/journal.pcbi.1003630)
Supplement: Figure S7 — Median percentages of time spent above 500 cells/µL and median numbers of cycles over a 24 month follow-up. For the simulations of repeated administrations of IL-7, we assumed that injections might have reduced effects compared to the first one of (1-απ)% and (1-αμQ)%. (DOC) [file pcbi.1003630.s007.doc]

**Figure S7. Median percentages of time spent above 500 cells/μL and median numbers of cycles over a 24 month follow-up.** For the simulations of repeated administrations of IL-7, we assumed that injections might have reduced effects compared to the first one of (1-α_π_)% and (1-α_μQ_)%.
