# Supplementary material for: Quantifying and Predicting the Effect of Exogenous Interleukin-7 on CD4+T Cells in HIV-1 Infection
Source: PLoS Comput Biol. 2014 May 22;10(5):e1003630. doi: 10.1371/journal.pcbi.1003630 (PMC4031052; doi:10.1371/journal.pcbi.1003630)
Supplement: Table S1 — Estimates of model parameters for total CD4+ and CD4+Ki67+ T-cell dynamics in Study II (INSPIRE Study). Model 1: only the proliferation rate (π) is modified; Model 2: proliferation rate (π) and loss rate (μQ) of non-proliferating cells are modified; Model 3: proliferation rate and constant production rate (λ) are modified. All IL-7 effects underlined in grey were statistically significant at 0.05 level. Standard-errors are given between brackets. (DOC) [file pcbi.1003630.s008.doc]

**Table S1. Estimates of model parameters for total CD4^+^ and CD4^+^Ki67^+^ T-cell dynamics in Study II (INSPIRE Study). Model 1: only the proliferation rate (π) is modified; Model 2: proliferation rate (π) and loss rate (μ_Q_) of non-proliferating cells are modified; Model 3: proliferation rate and constant production rate (λ) are modified. All IL-7 effects underlined in grey were statistically significant at 0.05 level. Standard-errors are given between brackets.**

|  |  |  | **Model 1** | **Model 2** | **Model 3** |
| --- | --- | --- | --- | --- | --- |
| **Parameters** |  | **LCVa^$^** | **0.937** | **-0.173** | **-0.131** |
| **Production Rate** | before & during IL7 | | 1.768 (0.200) | 9.849 (2.126) | 6.074 (1.168) |
| **(λ, cells/day)** | after IL7 | 10 µg/kg | 1.768 | 9.849 | 8.855 (2.024) |
|  |  | 20 µg/kg | 1.768 | 9.849 | 9.984 (2.282) |
|  |  | 30 µg/kg | 1.768 | 9.849 | 11.26 (2.573) |
|  |  |  |  |  |  |
| **Proliferation Rate** | before & after IL7 | | 0.018 (0.003) | 0.027 (0.004) | 0.025 (0.004) |
| **(π, /day)** | during IL7 | 10 µg/kg | 0.072 (0.016) | 0.107 (0.026) | 0.100 (0.026) |
|  |  | 20 µg/kg | 0.090 (0.020) | 0.129 (0.032) | 0.122 (0.032) |
|  |  | 30 µg/kg | 0.113 (0.025) | 0.156 (0.039) | 0.148 (0.039) |
|  |  |  |  |  |  |
| **Loss rate of non-** | before & during IL7 | | 0.022 (0.003) | 0.061 (0.010) | 0.045 (0.006) |
| **proliferating cells** | after IL7 | 10 µg/kg | 0.022 | 0.049 (0.008) | 0.045 |
| **(µ_Q_, /day)** |  | 20 µg/kg | 0.022 | 0.046 (0.008) | 0.045 |
|  |  | 30 µg/kg | 0.022 | 0.044 (0.007) | 0.045 |
|  |  |  |  |  |  |
| **Loss rate of proliferating cells (µ_P_, /day)** |  |  | 0.039 (0.015) | 0.070 (0.031) | 0.066 (0.028) |
|  |  |  |  |  |  |
| **Reversion rate to quiescent state (ρ, /day)** |  |  | 0.879 (0.116) | 1.213 (0.173) | 1.142 (0.156) |
| **σ_λ_*** |  |  | 0.208 (0.066) | -0.205 (0.066) | 0.206 (0.066) |
| **σ_ρ_*** |  |  | 0.380 (0.144) | 0.396 (0.165) | 0.379 (0.164) |
| ^$^ Likelihood cross-validated criteria: lower value indicated better model  * Standard-deviation of random effect. Note that the random effects were on the log-transformed parameter and not on the natural scale | | | | | |
