# Supplementary material for: Quantifying and Predicting the Effect of Exogenous Interleukin-7 on CD4+T Cells in HIV-1 Infection
Source: PLoS Comput Biol. 2014 May 22;10(5):e1003630. doi: 10.1371/journal.pcbi.1003630 (PMC4031052; doi:10.1371/journal.pcbi.1003630)
Supplement: Table S2 — Estimates of model parameters for total CD4+ and CD4+Ki67+ T-cell dynamics in Study I (rh-IL-7 study). Model 1: only the proliferation rate (π) is modified; Model 2: proliferation rate (π) and loss rate (μQ) of non-proliferating cells are modified; Model 3: proliferation rate and constant production rate (λ) are modified. All IL-7 effects underlined in grey were statistically significant at 0.05 level. Standard-errors are given between brackets. (DOC) [file pcbi.1003630.s009.doc]

**Table S2. Estimates of model parameters for total CD4^+^ and CD4^+^Ki67^+^ T-cell dynamics in Study I (rh-IL-7 study).** Model 1: only the proliferation rate (π) is modified; Model 2: proliferation rate (π) and loss rate (μ_Q_) of non-proliferating cells are modified; Model 3: proliferation rate and constant production rate (λ) are modified. All IL-7 effects underlined in grey were statistically significant at 0.05 level. Standard-errors are given between brackets.

|  |  |  | **Model 1** | **Model 2** | **Model 3** |
| --- | --- | --- | --- | --- | --- |
| **Parameters** |  | **LCVa^$^** | **3.814** | **2.644** | **2.491** |
| **Production Rate** | before & during IL7 | | 2.883 (1.285) | 11.331 (5.503) | 8.010 (3.898) |
| **(λ, cells/day)** | after IL7 | 3 µg/kg | 2.883 | 11.331 | 9.768 (5.560) |
|  |  | 10 µg/kg | 2.883 | 11.331 | 13.356 (7.603) |
|  |  |  |  |  |  |
| **Proliferation Rate** | before & after IL7 | | 0.046 (0.015) | 0.046 (0.033) | 0.048 (0.027) |
| **(π, /day)** | during IL7 | 3 µg/kg | 0.252 (0.152) | 0.225 (0.245) | 0.225 (0.209) |
|  |  | 10 µg/kg | 0.504 (0.304) | 0.438 (0.478) | 0.478 (0.443) |
|  |  |  |  |  |  |
| **Loss rate of non-** | before & during IL7 | | 0.020 (0.006) | 0.068 (0.025) | 0.051 (0.019) |
| **proliferating cells** | after IL7 | 3 µg/kg | 0.020 | 0.057 (0.024) | 0.051 |
| **(µ_Q_, /day)** |  | 10 µg/kg | 0.020 | 0.048 (0.021) | 0.051 |
|  |  |  |  |  |  |
| **Loss rate of proliferating cells (µ_P_, /day)** |  |  | 0.428 (0.126) | 0.338 (0.137) | 0.360 (0.140) |
|  |  |  |  |  |  |
| **Reversion rate to quiescent state (ρ, /day)** |  |  | 0.629 (0.124) | 0.670 (0.033) | 0.671 (0.153) |
| **σ_λ_*** |  |  | -0.308 (0.141) | 0.308 (0.206) | 0.309 (0.194) |
| **σ_ρ_*** |  |  | 0.074 (0.053) | -0.178 (0.187) | 0.145 (0.166) |
| ^$^ Likelihood cross-validated criteria: lower value indicated better model  * Standard-deviation of random effect. Note that the random effects were on the log-transformed parameter and not on the natural scale | | | | | |
