# Supplementary material for: Quantifying and Predicting the Effect of Exogenous Interleukin-7 on CD4+T Cells in HIV-1 Infection
Source: PLoS Comput Biol. 2014 May 22;10(5):e1003630. doi: 10.1371/journal.pcbi.1003630 (PMC4031052; doi:10.1371/journal.pcbi.1003630)
Supplement: Table S3 — Estimates of model parameters for naive CD4+ and naive CD4+Ki67+ T-cell dynamics in Study II (INSPIRE Study). Model 1: only the proliferation rate (πN) is modified; Model 2: proliferation rate (πN) and loss rate (μQ N) of non-proliferating cells are modified; Model 3: proliferation rate and constant production rate (λN) are modified. All IL-7 effects underlined in grey were statistically significant at 0.05 level. Standard-errors are given between brackets. (DOC) [file pcbi.1003630.s010.doc]

**Table S3. Estimates of model parameters for naive CD4^+^ and naive CD4^+^Ki67^+^ T-cell dynamics in Study II (INSPIRE Study). Model 1: only the proliferation rate (π^N^) is modified; Model 2: proliferation rate (π^N^ ) and loss rate (μ_Q_^N^) of non-proliferating cells are modified; Model 3: proliferation rate and constant production rate (λ^N^) are modified. All IL-7 effects underlined in grey were statistically significant at 0.05 level. Standard-errors are given between brackets.**

|  |  |  | **Model 1** | **Model 2** | **Model 3** |
| --- | --- | --- | --- | --- | --- |
| **Parameters** |  | **LCVa^$^** | **1.830** | **1.760** | **1.705** |
| **Production Rate** | before & during IL7 | | 1.308 (0.295) | 3.053 (1.440) | 3.600 (1.518) |
| **(λ^N^, cells/day)** | after IL7 | 10 µg/kg | 1.308 | 3.053 | 4.904 (2.883) |
|  |  | 20 µg/kg | 1.308 | 3.053 | 6.619 (3.347) |
|  |  | 30 µg/kg | 1.308 | 3.053 | 8.935 (3.885) |
|  |  |  |  |  |  |
| **Proliferation Rate** | before & after IL7 | | 0.003 (0.001) | 0.003 (0.001) | 0.004 (0.001) |
| **(π^N^, /day)** | during IL7 | 10 µg/kg | 0.062 (0.032) | 0.067 (0.032) | 0.068 (0.003) |
|  |  | 20 µg/kg | 0.092 (0.047) | 0.100 (0.048) | 0.105 (0.003) |
|  |  | 30 µg/kg | 0.136 (0.070) | 0.148 (0.071) | 0.161 (0.004) |
|  |  |  |  |  |  |
| **Loss rate of non-** | before & during IL7 | | 0.019 (0.004) | 0.043 (0.018) | 0.050 (0.019) |
| **proliferating cells** | after IL7 | 10 µg/kg | 0.019 | 0.030 (0.015) | 0.050 |
| **(µ_Q_^N^, /day)** |  | 20 µg/kg | 0.019 | 0.028 (0.014) | 0.050 |
|  |  | 30 µg/kg | 0.019 | 0.027 (0.013) | 0.050 |
|  |  |  |  |  |  |
| **Loss rate of proliferating cells (µ_P_^N^, /day)** |  |  | 0.081 (0.040) | 0.077 (0.038) | 0.078 (0.039) |
|  |  |  |  |  |  |
| **Reversion rate to quiescent state (ρ^N^, /day)** |  |  | 1.050 (0.217) | 1.109 (0.233) | 1.199 (0.255) |
| **σ_λ_*** |  |  | 0.530 (0.141) | 0.565 (0.160) | 0.523 (0.132) |
| **σ_ρ_*** |  |  | 0.434 (0.288) | -0.459 (0.278) | 0.458 (0.308) |
| ^$^ Likelihood cross-validated criteria: lower value indicated better model | | | | | |
| * Standard-deviation of random effect. Note that the random effects were on the log-transformed parameter and not on the natural scale | | | | | |
