# Supplementary material for: Quantifying and Predicting the Effect of Exogenous Interleukin-7 on CD4+T Cells in HIV-1 Infection
Source: PLoS Comput Biol. 2014 May 22;10(5):e1003630. doi: 10.1371/journal.pcbi.1003630 (PMC4031052; doi:10.1371/journal.pcbi.1003630)
Supplement: Table S4 — Percentage of time spent above 500 cells/µL, number and median time between cycles according to various scenarios. (DOC) [file pcbi.1003630.s011.doc]

Table S4. Percentage of time spent above 500 cells/μL, number and median time between cycles according to various scenarios.

| Scenario name | α_π_ (%) | α_μQ_ (%) | T_full (Days)_ | T_end (Days)_ | Median percentage of time spent above 500 cells/µL  ([IQR]* ) | Median number of cycles  ([IQR] ) | Median time between cycles ([IQR] Days) | |
| --- | --- | --- | --- | --- | --- | --- | --- | --- |
| ref. | No re-injection | | | | 11.5  [ 7.0; 16.0] | 1 | | - |
| A - 1 | 100 | 100 | 270 | 731 | 85.4  [83.9; 90.6] | 2  [1; 2] | | 360  [180; 450] |
| A - 2 |  |  | 90 | 365 | 68.9  [68.5; 73.7] | 3  [2; 3] | | 180  [180; 270] |
| B - 1 | 50 | 100 | 270 | 731 | 84.8  [79.6; 89.7] | 2  [1; 2] | | 360  [180; 450] |
| B - 2 |  |  | 90 | 365 | 66.2  [55.7; 80.0] | 3  [2; 3] | | 180  [90; 270] |
| C - 1 | 0 | 100 | 270 | 731 | 78.1  [75.2; 80.8] | 2  [1; 2] | | 180  [90; 450] |
| C - 2 |  |  | 90 | 365 | 60.9  [59.0; 75.2] | 3  [2; 4] | | 180  [90; 180] |
| D - 1 | 100 | 50 | 270 | 731 | 77.0  [71.4; 85.6] | 2  [1; 3] | | 270  [180; 360] |
| D - 2 |  |  | 90 | 365 | 65.7  [59.6; 68.7] | 4  [3; 4] | | 180  [90; 180] |
| E - 1 | 100 | 0 | 270 | 731 | 59.4  [55.5; 63.1] | 4  [2; 5] | | 90  [90; 180] |
| E - 2 |  |  | 90 | 365 | 49.7  [40.2; 53.8] | 6  [5; 6] | | 90  [90; 90] |
| F - 1 | 0 | 50 | 270 | 731 | 60.5  [54.6; 67.2] | 3  [1; 4] | | 180  [90; 360] |
| F - 2 |  |  | 90 | 365 | 19.6  [7.0; 29.8] | 5  [3; 7] | | 90  [90; 90] |
| G - 1 | 50 | 0 | 270 | 731 | 40.6  [25.6; 44.0] | 6  [3; 7] | | 90  [90; 90] |
| G - 2 |  |  | 90 | 365 | 14.9  [14.2; 20.9] | 6  [5; 7] | | 90  [90; 90] |

* InterQuartile Range
